# Isolation of *Catharanthus roseus* (L.) G. Don Nuclei and Measurement of Rate of *Tryptophan decarboxylase* Gene Transcription Using Nuclear Run-On Transcription Assay

**DOI:** 10.1371/journal.pone.0127892

**Published:** 2015-05-29

**Authors:** Santosh Kumar, Sabhyata Bhatia

**Affiliations:** National Institute of Plant Genome Research, Aruna Asaf Ali Marg, PO Box 10531, New Delhi, 110067, India; Mount Allison University, CANADA

## Abstract

**Background:**

An accurate assessment of transcription ‘rate’ is often desired to describe the promoter activity. In plants, isolation of transcriptionally active nuclei and their subsequent use in nuclear run-on assays has been challenging and therefore limit an accurate measurement of gene transcription ‘rate’. *Catharanthus roseus* has emerged as a model medicinal plant as it exhibits an unsurpassed spectrum of chemodiversity, producing over 130 alkaloids through the terpenoid indole alkaloid (TIA) pathway and therefore serves as a ‘molecular hub’ to understand gene expression profiles.

**Results:**

The protocols presented here streamline, adapt and optimize the existing methods of nuclear run-on assay for use in *C*. *roseus*. Here, we fully describe all the steps to isolate transcriptionally active nuclei from *C*. *roseus* leaves and utilize them to perform nuclear run-on transcription assay. Nuclei isolated by this method transcribed at a level consistent with their response to external stimuli, as transcription rate of TDC gene was found to be higher in response to external stimuli i.e. when seedlings were subjected to UV-B light or to methyl jasmonate (MeJA). However, the relative transcript abundance measured parallel through qRT-PCR was found to be inconsistent with the synthesis rate indicating that some post transcriptional events might have a role in transcript stability in response to stimuli.

**Conclusions:**

Our study provides an optimized, efficient and inexpensive method of isolation of intact nuclei and nuclear ‘run-on’ transcription assay to carry out *in-situ* measurement of gene transcription rate in *Catharanthus roseus*. This would be valuable in investigating the transcriptional and post transcriptional response of other TIA pathway genes in *C*. *roseus*. Isolated nuclei may also provide a resource that could be used for performing the chip assay as well as serve as the source of nuclear proteins for *in-vitro* EMSA studies. Moreover, nascent nuclear run-on transcript could be further subjected to RNA-Seq for global nuclear run-on assay (GNRO-Seq) for genome wide *in-situ* measurement of transcription rate of plant genes.

## Introduction

**Fig 1:**
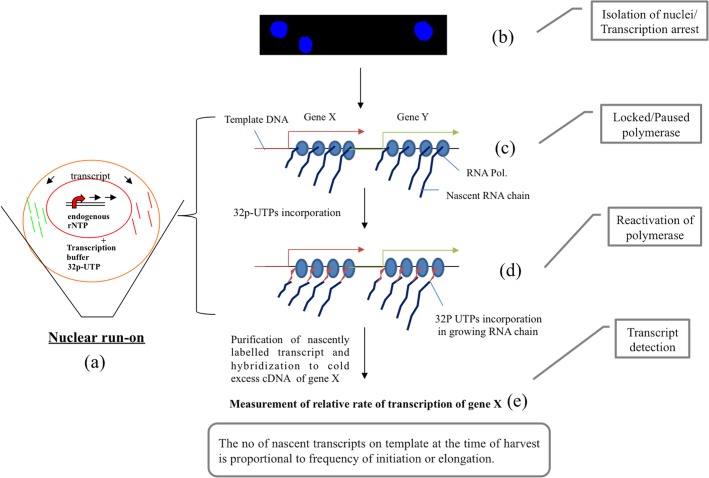
Schematic representation of nuclear run-on assay and mechanism of incorporation of radiolabelled ^32^P-UTP and visualization of relative rate of transcription (Partially adapted from Weber et al., 1977). (a) *in-vitro* transcription (b) paused polymerase with cognate templates (c) reactivation of polymerase and incorporation of labeled UTPs (d) measurement of relative transcription rate.

Gene expression is, in general, inferred by measuring transcript abundance. Methods such as RNA gel blotting (Northern blotting), semi-quantitative RT–PCR and real-time quantitative PCR [1–3] provide information about the steady-state accumulation of a given RNA transcript at a given time point. However, the detection of transcript accumulation by these methods is only an indirect estimate of gene activity or induction, because the relative amount of specific mRNA depends on the transcription rate and the mechanisms that stabilize or destabilize the resulting transcript. Methods to assay transcription rate contribute important information regarding the first kinetics in the central dogma of molecular biology. One such method which helps to accurately assess the rate of transcription is the nuclear run-on assay which provides an *in- situ* measure of transcription rate of specific genes in transcriptionally active nuclei. The difference between monitoring gene expression by the nuclear run-on assay versus most other assays (Northern blot analysis and/or qRT-PCR analysis) is that the nuclear run-on assay provides an *in situ* measure of the frequency of transcription initiation and is largely independent of the effects of kinetics of degradation. Briefly, nuclear run-on assay begins with intact nuclei isolated from fresh tissue harvested from a given developmental state or after a specific treatment. Upon purification of nuclei (Fig 1B), transcription is arrested with RNA polymerases locked/paused in their transcriptionally active conformation, in association with their cognate templates (Fig 1C). Nuclei are then incubated for a short time in transcription assay buffer with nucleoside triphosphate (NTPs) and radiolabelled uridine 5’-triphosphate (UTPs) (Fig 1A). The radiolabeled nucleotide gets incorporated into transcripts upon reactivation of engaged/locked polymerase for continuing synthesis of RNA (Fig 1D) which can be then hybridized to filter-bound, cold, excess DNA probes representing genes of interest (Fig 1E). Hybridization signals represent the amount of transcript produced in the isolated nuclei—a direct reflection of the rate of transcription [4].

Parallel measurements of transcription rate and steady-state level of specific transcript allows for estimation of transcript decay. There are many cases in which nuclear run-on assays directly measured the transcription rate and have allowed one to reconcile discrepancies between transcription rates and transcript abundance [5–7]. However in plants, isolation of transcriptionally active nuclei and their subsequent use in nuclear run-on assays has been challenging and therefore limited. The plant species in which it has been possible to isolate transcriptionally active nuclei and subsequently use them in nuclear run-on assays include pea [8–11]; rice [12]; petunia [13]; soybean [14, 15] and *A*. *thaliana* [16–19].

*Catharanthus roseus* (Madagascar periwinkle) is widely known for its pharmacologically important alkaloids such as the anti-cancer compounds vinblastine and vincristine. The plant exhibits an unsurpassed spectrum of chemodiversity as it produces over 130 alkaloids through the terpenoid indole alkaloid (TIA) pathway [20]. This is a multistep and very complex pathway involving the regulated expression of many genes, several of which are affected by biotic and abiotic factors. For example, *Tryptophan decarboxylase* (TDC), one of the most important genes of TIA pathway is developmentally regulated gene [21, 22] and it also strongly responds to a group of chemical or fungal elicitors and UV-B light [23–26]. TDC enzyme converts tryptophan into tryptamine, and is a key enzyme in the TIA biosynthetic pathway due to its position on the interface of primary and secondary metabolism. Therefore, a study of the transcription kinetics of the TDC would provide an opportunity to understand the molecular basis of gene expression in the TIA pathway. However, there are no reports available that document the successful preparation of transcriptionally active nuclei and their subsequent use in run-on assay in *C*. *roseus*. Here, we fully describe all the steps to perform nuclear run-on transcription assay from intact transcriptionally active nuclei isolated from leaves of model medicinal plant *C*. *roseus*. The present method may be used to accurately measure the gene activity and or its induction which in turn would unravel the transcriptional regulatory mechanism occurring in different tissues, not only of *C*. *roseus* but of various other plant species. Moreover, availability of this protocol for isolation of intact nuclei would greatly facilitate an aid in performing experiments such as the isolation of nuclear proteins and chromatin immune-precipitation assays.

## Materials and Methods

### Plant material and treatments

Seeds of *Catharanthus Roseus* var Nirmal were germinated in pots containing peat:vermiculite:perlite (1:1:1) and kept in a growth chamber at 26°C under a 16-h photoperiod with light intensity of 380+/-25mmole^-2^s^-1^ generated by an overhead fluorescent lamp. Four week-old seedlings were used for treatments.

### Methyl jasmonate (MeJA) and UV-B light treatment

0.5 mM MeJA was employed to treat plants. The seedlings were taken out of the growth chamber and immersed in MeJA solution for 30 sec. The plants were then labeled and covered with a transparent plastic lid before being moved back into the growth chamber. The UV-B light (λ = 312 nm) treatment was performed by irradiating the seedlings in pots for 2 min in the UV light chamber. Plants were then labeled and moved back into the growth chamber. Shoot tips from 6 to 9 treated plants including1- 4 true leaves, were excised 6h after MeJA treatment and UV treatments and combined as one biological replicate. Three biological replicates were analyzed for each treatment. Treated tissue samples were harvested and immediately frozen in liquid nitrogen and kept at -80°C for further analyses.

### Reagents and buffers

Hexylene glycol (+/^_^ 2-methyl-2,4-pentadiol; Sigma-Aldrich), PIPES-KOH (Sigma-Aldrich), MgCl_2_, 2-Mercaptoethanol (Sigma-Aldrich), Glycerol, Triton X-100 (Sigma-Aldrich), Sucrose, Percoll (Sigma-Aldrich), Tris–HCl, pH 7.8, Ribonuclease inhibitor (RNAseIn; Promega), CTP, GTP and ATP (Epicenter Inc.), Uridine 5’-[α-^32^P] triphosphate, triethylammonium salt (3,000 Ci mmol^-^1), (BARC/India), Phenol/chloroform/octanol reagent [25:24:1] (pH 7.6), yeast tRNA (Sigma-Aldrich), ethanol, NaOH, DNase I (NEB), Ammonium acetate (Sigma-Aldrich), DAPI (Sigma-Aldrich) and KOH.

#### Extraction buffer (1X)

2.0 M hexylene glycol (2-methyl-2,4-pentandiol), 20 mM PIPES-KOH (pH 7.0), 10 mM MgCl_2_ and 5 mM 2-mercaptoethanol.

#### Gradient buffer (1X)

0.5 M hexylene glycol, 5 mM PIPES-KOH (pH 7.0), 10 mM MgCl_2_, 5 mM 2-mercaptoethanol and 1% Triton X-100.

#### Nuclei storage buffer

50 mM Tris–HCl (pH 7.8), 10 mM 2-mercaptoethanol, 20% glycerol, 5 mM MgCl_2_ and 0.44 M sucrose.

#### Transcription assay buffer (10X)

250mM Tris–HCl (pH 7.8), 375mM NH_4_Cl, 50mM MgCl_2_ and 50% (v/v) glycerol.

#### Termination buffer

7.5 M urea, 0.5% SDS, 20 mM EDTA (pH 7.5) and 100 mM LiCl

SSC (20X): 3.0 M NaCl and 0.3 M sodium citrate (pH 7.0)

### Isolation of *C*. *roseus* nuclei

#### Percoll gradient assembly

80% and 30% percoll suspension was prepared by mixing 100% percoll with appropriate amounts of 5x gradient buffer and water to achieve a final concentration of 1x gradient buffer. 6 ml of 30% percoll solution in 1x gradient buffer was added to a 50 ml round bottom culture tube. Using pasture pipette, 6 ml of 80% percoll solution was gently added to the bottom of the test tube by passing the pipette through the 30% layer and slowly expelling the solution into the very bottom of the test tube. Complete gradient assembly was done at 4°C.

#### Nuclei isolation

Intact nuclei were isolated using the procedure of Folta and Kaufman (2006) [19] with some modifications. Six grams (6g) of *C*. *roseus* leaf tissue in liquid nitrogen was homogenized using a pestle and mortar. The homogenized powdered tissue was suspended in 30 ml of 1X extraction buffer **(**2.0 M hexylene glycol), 20 mM PIPES-KOH (pH 7.0), 10 mM MgCl_2_ and 5 mM 2-mercaptoethanol). 3–5 layers of cheesecloth, soaked in extraction buffer, were placed over 50 ml culture tubes. The homogenate was slowly decanted through the cheesecloth filter and the resulting extract was gently stirred over ice using a magnetic stirrer plate. Then 10% triton-X-100 was added gently (drop wise) to the solution until the final concentration was 1%. The filtered plant extract was gently pipetted onto the top of the percoll gradient assembled in 50ml culture tube. The gradient was then centrifuged at 2,000x g for 30 min at 4°C. Following centrifugation, the nuclei were seen as a dark band at the interface of the 80% and 30% layers. The top layer of extraction buffer was gently aspirated out using a 25 ml glass pipette followed by collection of nuclei from the 30%-80% interface using a pasture pipette. To the enriched nuclei, 1x gradient buffer was added to bring the total volume to 10 ml, and then the solution was under laid with 6ml of 30% percoll. The gradient was again centrifuged at 2,000x g for 10 min at 4°C and supernatant containing gradient buffer and percoll was gently discarded. Top layer of pellet was resuspended in 1x gradient buffer (about 1.5ml) and transferred to a microcentrifuge tube followed by centrifugation at 900x g for 5 min. The nuclei pellet was then dissolved in 500μl of nuclei storage buffer. Nuclear aliquots were made and stored at -80°C until use.

#### DAPI staining

Nuclei sample was equilibrated briefly with phosphate-buffer saline (PBS) and stained with 300μl of diluted DAPI (300nM) solution on a glass slide. Sample was incubated for 5 min at room temperature and then rinsed several times in PBS. Excess buffer was drained off from under the cover slip and the slide was mounted and nuclei visualized under fluorescent microscope (Nikon) with appropriate filters.

### Run-on transcription assay

For in-vitro synthesis of radiolabelled transcript with intact isolated nuclei, approximately 10^6^ nuclei in 50μl of nuclei storage buffer were allowed to pre-incubate for 10 min with 20 units RNAsin (Promega) at 30°C. Following pre-incubation, a pre-warmed transcription assay reaction mixture was added to each sample. This reaction mixture contained 10μl of 10x transcription buffer, 5μl of 100 mM CTP, GTP and ATP, 10μl of ^32^P-UTP, 50μl nuclei in storage buffer and 15μl of water to make total reaction 100μl and reaction was allowed to proceed at 30°C for 30 min. Reaction was terminated by using 10U DNase I and incubated at 30°C for 10 min followed by addition of 200μl of termination buffer and addition of 300μl of phenol-chloroform-octanol reagent. The sample was vortexed for 1 min followed by centrifugation at 20,000xg for 5 min at room temperature. Top aqueous fraction was transferred to a clean microcentrifuge tube and to this 1/10 volume 7.5M ammonium acetate (pH 7.0) and 100μg of yeast tRNA along with 2 volumes of ice-cold ethanol was added. Then the sample was placed on ice for 10 min followed by centrifugation at 2,000x g for 10 min. The supernatant was discarded and the pellet was dissolved in 500μl of hybridization buffer and stored at -80°C for further use in hybridization.

#### Blot preparation, hybridization and autoradiography

The gene sequences used for expression analysis either via run-on assay or through qRT-PCR analysis were *Tryptophan decarboxylase* gene (TDC: M25151.1) [27] as a target and the set of reference gene 40S ribosomal subunit 9 (RPS9: Caros004092.1/AJ749993), SAND (Caros010066.1), N2227 (Caros011588.1) [28,29] along with Monovinsinine 19-hydroxy-O-acetyltransferase (MAT: AF253415) [30] as a negative control. The complimentary DNA (cDNA) fragments of respective genes were amplified using gene specific primers targeted the coding regions excluding the 5’ and 3’—UTRs regions of gene (S1 Table). PCR was performed in a 20μl reaction volume. For each primer set following components were added: 100ng cDNA, 1μl of 10x PCR buffer, 1μl dNTPs and 1μl pf each forward and reverse primers, 0.2μl of Titanium Tag and milliQ to make final volume up to 20μl. The PCR condition used for amplification was an initial denaturation at 95°C for 2 min followed by 35 cycle of denaturation at 95°C for 30 sec, annealing at 60°C for 30 sec and extension at 72°C. PCR amplicons were electrophoresed on agarose gel, eluted and confirmed by Sanger sequencing (ABI) For dot blot analysis at least 1μg of PCR amplified DNA of TDC and RPS9 was denatured in 0.4 N NaOH in a total volume of 100μl at 65°C for 15 min, followed by the addition of 200 μl ice-cold 20X SSC. The total contents of each sample were transferred onto the nylon blotting filter (Hybond-N; GE Health Care) via a dot-blot manifold by applying a gentle vacuum. The dot blot wells were rinsed with 10X SSC. To the membrane, a small amount of DNA gel loading dye was added which allowed visualization of the immobilized DNA on the membrane filter. Immobilized DNA was UV-crosslinked to the membrane followed by incubation with a pre-hybridization solution (Miracle-Hyb Buffer, Stratagene USA).

Southern blotting was also performed for which 10μg of PCR amplified DNA of TDC and reference genes (RPS9, N2227 and SAND) along with MAT was electrophoresed on 1% agarose gel followed by denaturation in 1.5 M NaCl, 0.5 M NaOH for 30 min with constant gentle agitation. After denaturation, the gel was treated with neutralization buffer (0.5 M Tris-Cl pH 7.4, 1.5 M NaCl) for 30 min. The gel was finally soaked in neutral transfer buffer (10X SSC) for 20 min prior to the transfer of DNA to the membrane which was carried out overnight at room temperature using 10X SSC as transfer buffer. The DNA was fixed to the membrane in the UV cross-linker.

For hybridization, both the membranes with the immobilized DNAs were incubated at 60°C for 2–3h in prehybridization buffer. The total radio labelled run-on transcripts in hybridization buffer (Miracle-Hyb buffer, Stratagene USA) were denatured by heating at 95°C for 5 min and immediately placing on ice for 5 min. This was added to the prehybridization solution containing the membranes and hybridization was carried out for 16 h at 60°C. For washing, the hybridization solution was discarded and the membranes were washed twice with 2X SSC and 0.1% SDS (w/v) at room temperature followed by one wash in pre-warmed 0.2X SSC and 0.1% SDS at 60°C for 15 min. During the washing, care was taken to prevent drying of the labeled membranes.

For autoradiography, the semi-dried membrane was exposed to hyper screen (Amersham, USA) in a cassette and was kept at room temperature for 1–12 h (depending on radioactivity counts). Radioactive signals were detected with the help of a high resolution scanner (Typhoon, Amersham, USA). The band intensities were quantified with Quantyone software (BioRad)

### Quantitative real time PCR (qRT-PCR) analysis

Total RNA was extracted from the infiltrated leaves by LiCl method [31] and treated with 10 units of RNase free DNase 1 (Sigma) to remove DNA contamination. Two to five micrograms of total RNA was subjected to first strand synthesis using Power Script Reverse Transcriptase according to the manufacturer’s procedure using oligo (dT) primer. qRT-PCR was conducted as have been described previously [32]. PCR amplification was conducted using one-tenth of the reaction as a template. Primer pairs used for qRT-PCR analysis of different genes is mentioned in S1 Table.

## Results

### Isolation of nuclei from *C*. *roseus*

**Fig 2:**
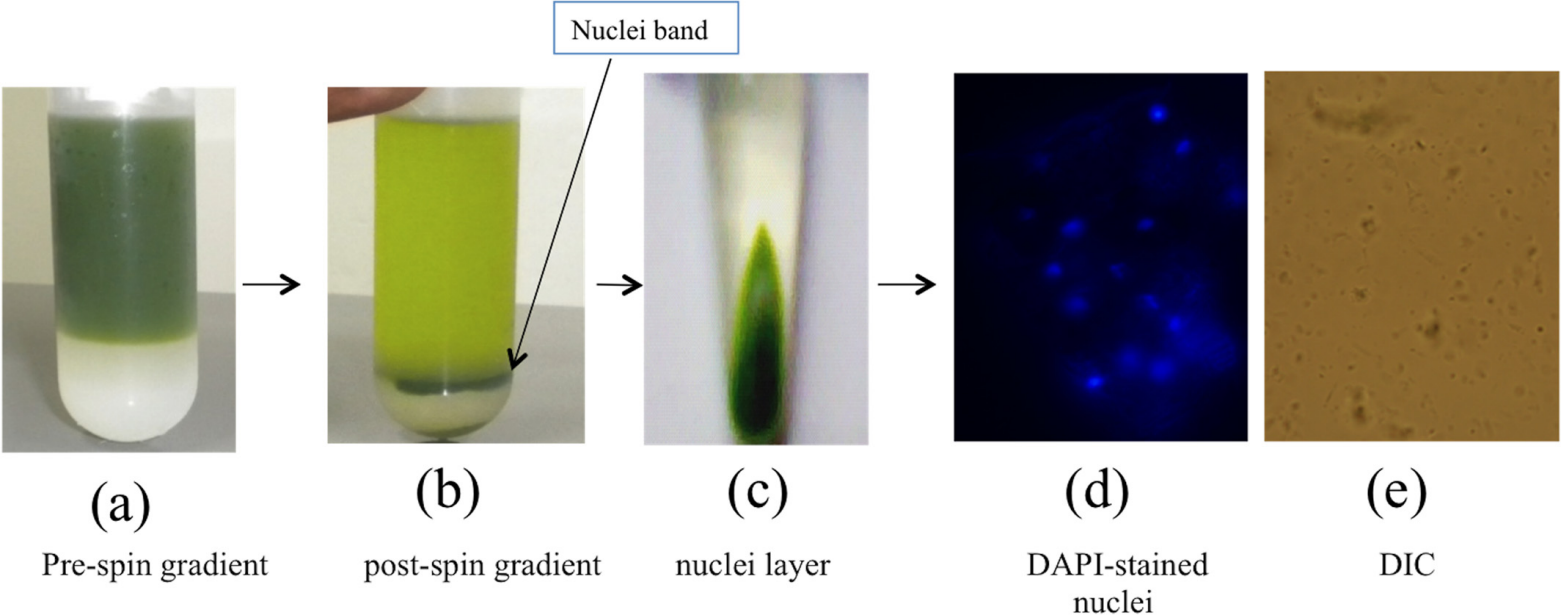
Overview of isolation of transcriptionally active nuclei from *C*. *roseus* accessions (a) pre-spin gradient (b) post-spin gradient (c) nuclei layer (d) DAPI stained nuclei (e) Differential interference contrast (DIC) view of nuclei.

Transcriptionally active nuclei were isolated from leaf samples harvested from *C*. *roseus* grown in the NIPGR fields. Isolation of nuclei was based on fractionation using percoll gradient centrifugation. Fresh leaf samples were ground to fine powder in liquid nitrogen and suspended in extraction buffer. The homogenate was filtered and layered onto the top of 30%–80% percoll gradient as shown in Fig 2A. This was centrifuged at 2000x g for 30 min at 4°C after which the nuclei band was visible as a dark layer at the interface of 30% and 80% gradient (Fig 2B). The nuclei band was collected into an eppendorf tube using a Pasteur pipette and centrifuged at 800x g for 5 min at 4°C. A pellet was obtained which was clearly separated into three layers (Fig 2C). The lowest layer was white and consisted mostly of starch, the middle one was the green debris layer whereas the top light grey layer was the one enriched with nuclei (Fig 2C). The nuclei enriched layer was further dissolved in nuclei storage buffer. DAPI staining was performed to confirm the presence of intact nuclei (blue; round and/or oval shape) as shown in Fig 2D and dot spot in DIC.

### Assessment of ^32^P UTP incorporation and transcriptional activity in isolated nuclei

**Fig 3:**
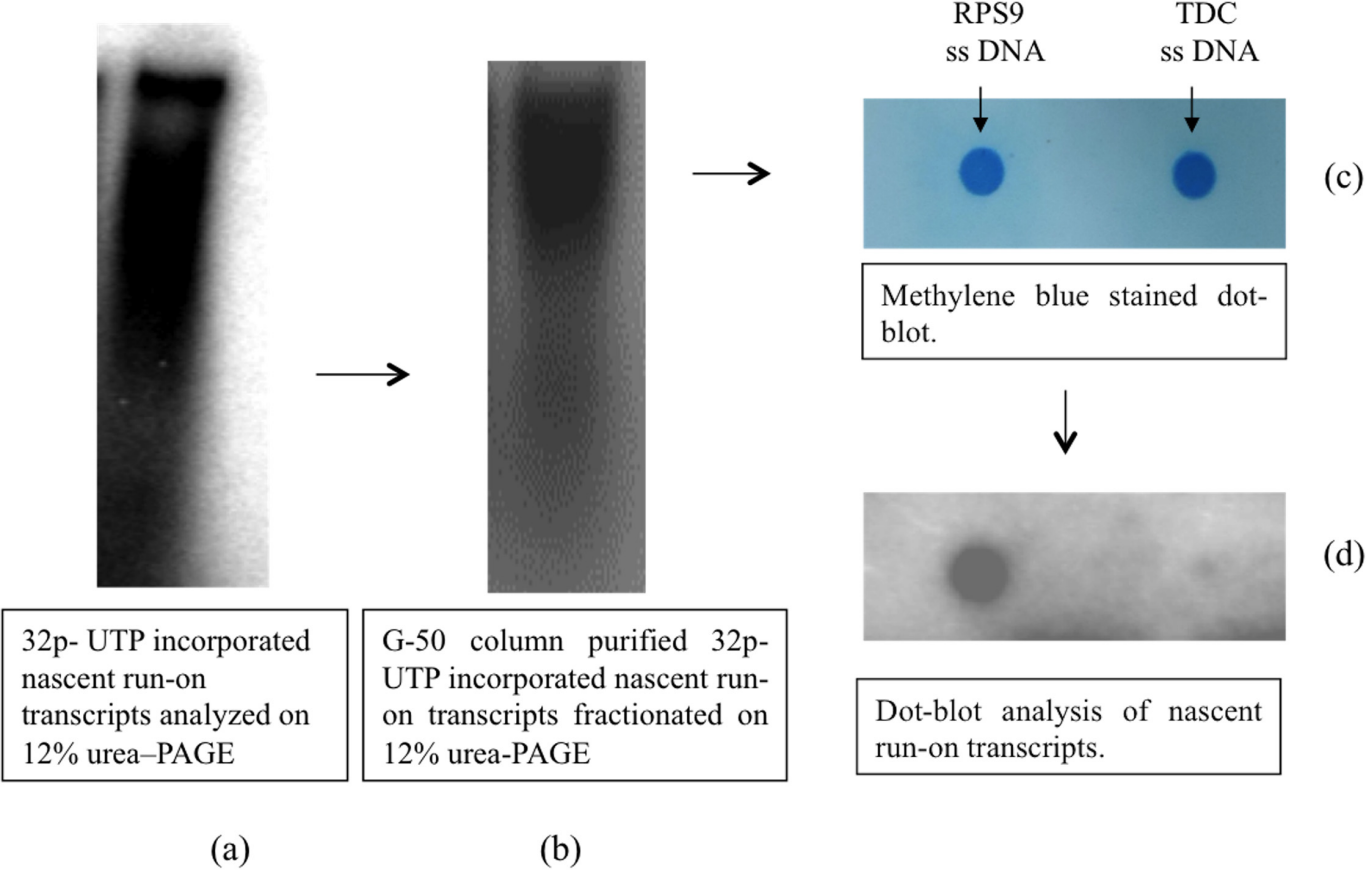
Standardization of nuclear run-on assay (a) ^32^P UTP incorporated total transcripts from *C*. *roseus* accessions analyzed on 12% urea–PAGE gel, (b) ^32^P UTP incorporated total transcripts purified on a G-50 purification column and fractionated on 12% urea-PAGE gel, (c) Methylene blue stained dot-blot of RPS9 and TDC (d) dot-blot after being probed with nascently synthesized radiolabelled run-on transcripts.

A standardization of the nuclear run-on assay was carried out using dot-blot methodology to confirm that nuclei were intact and transcriptionally active (Fig 3). For this, approximately 10^6^ nuclei were taken and run-on assay was performed by incubating them in presence of nucleoside triphosphate with one of them being radiolabelled (typically UTP), in an appropriate buffer. Here, the engaged polymerase synthesizes radiolabeled transcripts, which were purified and electrophoresed on 12% urea-PAGE gels followed by autoradiography. The cDNA like smears obtained before and after G50 column purification of run-on transcripts indicated successful incorporation of UTP in the wide size range of nuclear transcripts (Fig 3A and 3B). Next, the PCR amplified DNA of target gene (TDC) and constitutive expressing RPS9 gene were dot-blotted and fixed onto a membrane (Fig 3C). This blot was hybridized with the purified nascently synthesized UTP labeled nuclear transcripts. Autoradiography revealed a clear dark spot with RPS9 (positive control) thereby confirming that the isolated nuclei were intact and transcriptionally active (Fig 3D). However, an extremely faint signal against the TDC gene was obtained indicating very low basal expression of TDC gene.

This might be due to that TIA biosynthesis is developmentally regulated-preferential occurring in young leaf tissues [21, 22]. Therefore, ‘run-on’ transcription assay was further demonstrated with young leaf tissue (1–2 leaf pair) along with modification in reaction setup and also by analyzing the expression of TDC gene after induction by treatment with UV-B and MeJA.

### Transcription of TDC gene in response to UV-B light and MeJA

*C*. *roseus* TDC gene transcription was monitored both at the level of transcript synthesis and at the level of mRNA abundance via ‘run-on’ transcription assay and qRT-PCR analysis respectively. The reference gene used in both analyse were RPS9, SAND, N2227 and MAT (used as a negative control) in nuclear run-on assay.

The *C*. *roseus* seedlings were subjected to UV-B and MeJA treatments as described in material and methods. Nuclei were isolated from the stress treated seedlings as well as the untreated seedlings and ‘run-on’ transcription assay was performed with the following modifications to the earlier protocol: (1) The amount of nuclei was doubled with a proportionate increase in radiolabeled rUTPs (2) addition of a threshold concentration (1/100^th^ of rNTPs) of cold UTPs to the transcription assay buffer. Nuclear ‘run-on’ nascent transcript from both control and treated sample were then hybridized with respective blots harboring PCR amplified, denatured cDNA reference genes (RPS9, SAND, N2227) and target (TDC) gene. Two pairs of blots were made for hybridization with each set of nuclear ‘run-on’ transcripts synthesized from 2 treated and 2 control samples.

**Fig 4:**
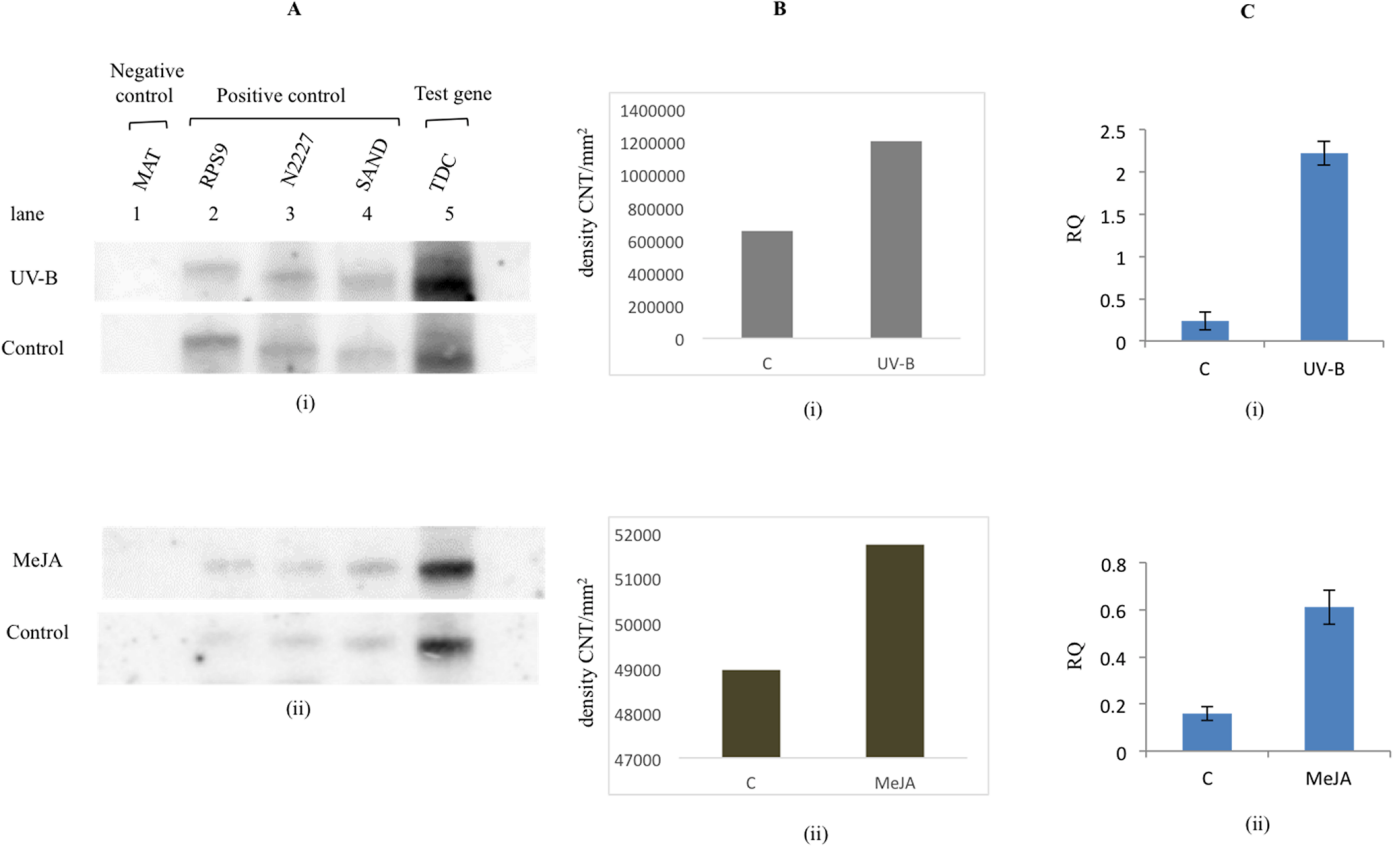
Measurement of rate of transcription of TDC gene and relative transcript abundance in *C*. *roseus*. (A) Autoradiogram of nuclear run-on assay in response to (i) UV-B light and (ii) MeJA. Reference genes were RPS9, SAND and N2227 (lane 2, 3 and 4), negative control gene was MAT (lane 1) and target gene was TDC (lane 5). (B) Relative quantification of TDC band intensity (Y-axis-density CNT/mm^**2**^) corresponding to panel (A) using QuantyOne software (BioRad). (C) Parallel quantification of relative transcript abundance of TDC gene as obtained by qRT-PCR in response to corresponding treatments depicted in panel (A) [Y-axis-relative quotient (RQ)].

The rate of TDC transcription in control and in response to stimuli was indicated by the respective band intensity (Fig 4. Panel A i and ii)] and relative quantification of corresponding band in Fig 4. Panel B i and ii. However, relative quantification of TDC transcript abundance in response to respective stimuli was depicted in Fig 4. Panel C i and ii. Through these experiments, differential rate of transcription of TDC gene under untreated and treated samples could be successfully observed. This revealed that the rate of TDC gene transcription was more than two fold higher in response to UV-B (Fig 4Ai and 4Bii) and there was slight induction in response to MeJA (Fig 4Ai and 4Bii) treatments as compared to control sample (Fig 4Ai). However, TDC transcript abundance in response to both MeJA and UV-B was not in proportion with the corresponding transcript synthesis rate. The TDC transcript abundance in response to UV-B and MeJA was found to be 10 fold and 3 fold higher respectively in comparison to control.

## Discussion

Studies of gene transcription, synthesis and processing of RNA and post-transcriptional gene silencing are enhanced by the availability of transcriptionally active nuclei [33–35]. Therefore, isolation of transcriptionally active nuclei is a fundamental first step towards the successful completion of transcription assays such as nuclear ‘run-on’. This adds an additional level of resolution to assay of steady-state transcript accumulation such as Northern blot, microarray or qRT-PCR analysis. In this study, we present a standardized protocol for the isolation of intact and transcriptionally active *C*. *roseus* nuclei and also successfully demonstrate the nuclear ‘run-on’ transcription assay. The protocol presented here for isolation of nuclei is a modification of the methods presented by Folta and Kaufman (2006). In animals, nuclei can be directly released by lysing the cell with nonionic detergents, such as Triton X-100 or Nonidet P-40, which destroy cellular membranes but leave nuclear membrane intact. However, unlike animal nuclei, plant nuclei can be released only after disruption of the cell wall and cellular membrane. This is commonly achieved by grinding the plant tissue in liquid nitrogen and/ or homogenizer. Following filtration of the homogenized tissue solution through Miracloth, which separates the subcellular particles from the tissue and cell fragments, the intact nucleus can be purified further by removing the chloroplast with nonionic detergents. Our results indicate that grinding the plant tissue in liquid nitrogen is an efficient means to fractionate plant cells and release intact nuclei. After cell disruption, keeping the homogenized tissue solution at a low temperature (0–4°C) with a sufficient concentration of beta-mercaptoethanol is important to inhibit activities of endogenous cytoplasmic enzymes. In the present protocol, we utilized a hexylene glycol-based extraction buffer rather than the Honda Buffer [36] which has been routinely utilized. In many earlier studies, percoll-density centrifugation had been employed to separate nuclei from cellular debris and organelles [37, 38] whereas other protocols implemented sucrose gradients [39]. Percoll is composed of 15–30 nm colloidal silicon particles coated with polyvinylpyrrolidone. It offers the advantage of being biologically inert and allows a clear concentration of nuclei at the high-density/low-density interface [17]. Furthermore, organelle contaminations such as plastids were lysed by using 0.5%- 1% of Triton X-100 [40]. Other protocols [41] have been found to work across multiple tissues/plants. Similarly the protocol reported here was also utilized for isolating nuclei from unrelated tissues such as the chickpea seedlings and nodules (unpublished results) thereby demonstrating its versatility for use in a wide range of plants/tissues.

Once the intact nuclei are available, the measurement of transcription rate is then performed by incubating the isolated nuclei in the presence of nucleoside triphosphates, including radiolabeled UTP, in an appropriate run-on assay buffer which would aid the engaged polymerase synthesizes radiolabeled transcripts. Therefore the ‘run-on’ transcription assay buffer is a critical solution in this assay. The assay buffer has to provide the proper ionic strength to maintain morphological and functional integrity of nuclei since insufficient concentrations of cations would result in aggregation or disintegration of the nuclei [42]. Our results indicate that replacement of monovalent cation such as NH_4_^+^, suggested in the protocol of Folta and Kaufman [19] with K^+^ ions in transcription buffer was preferable to maintain morphological and functional integrity of nuclei. Moreover, the pH of the transcription buffer also affects transcription activity of nuclei. Published reports indicate that the highest transcription activity occurs at pH 8, and transcriptional elongation is inhibited by 50% when the pH is reduced from 7.9 to 6.7 [43]. Moreover, data from transcription and RNA synthesis studies in bacteria and *Ehrlich* ascites cells suggest that ATP and GTP concentration may eventually determine rates of ribosomal RNA synthesis [44, 45]. On the basis of these concentrations, we have used the saturating concentration of rNTPs and observed that the threshold value of cold UTP in addition to radiolabelled UTP is critical for maintaining high levels of transcription by engaged RNA polymerase. Proper aeration of the transcription buffer is helpful in maintaining high levels of transcription in isolated nuclei. RNase inhibitors are commonly added to the transcription buffer and are helpful in addition to other reagents such as EDTA, DTT etc.

Next, for isolation and purification of high quality RNA transcripts, some protocols prefer to use TriZol reagent [46]. However, we found the use of phenol:chloroform:octanol reagents for isolation of RNA transcript and use of G-50 column for the purification of high quality RNA transcripts to be more effective. Further, in order to assess the incorporation of radiolabelled rUTPs, the simple method of electrophoresis on 12% denaturing PAGE gel was used. The clear cDNA like smears that appeared on PAGE indicated that the radiolabeled nucleotide gets incorporated into transcripts upon reactivation of all engaged/locked RNA polymerase for continuing synthesis of RNA. In addition to this, dot blot analysis also confirmed that the isolated nuclei were intact and transcriptionally active. However, earlier methods report the use of scintillation counter for assessing incorporation of radiolabeled rUTP. Hence the procedure used by us is simple, low cost and did not require the use of expensive equipments.

Furthermore, *C*. *roseus* TDC gene is known to developmentally regulated [21, 22] and it also regulated by external stimuli such as jasmonic acid [23–25] and UV-B [26]. Therefore, *C*. *roseus* nuclei isolated from young leaf tissue (1–2 leaf pair) was tested for induction of TDC gene synthesis using nuclear run-on transcription assay. TDC transcribed at a level consistent with its response to external stimuli, as transcription rate of TDC gene was found to be higher in response to both the treatments i.e. when seedlings were subjected to a short pulse of UV-B light or low concentration of methyl jasmonate (MeJA). The rate of TDC gene transcription was found to be induced in response to UV-B (Fig 4Ai and 4Bi) and MeJA (Fig 4Ai and 4Aii). However, TDC transcript abundance in response to both MeJA and UV-B was not consistent with the corresponding transcript synthesis rate. Whereas, the TDC transcript abundance in response to UV-B and MeJA was found to be approximately 10 fold and 3 fold higher respectively in comparison to control. This suggested that post transcriptional stability could be one of the likely reasons leading to TDC transcript abundance in response to stimuli. In several other plant species nuclear run-on assays have been used to infer an unexpected change in transcript accumulation that could not likely be attributed to transcription alone. For example, Hansen et al. [47] and Petracek et al. [48] observed that light-enhanced accumulation of ferredoxin 1 transcripts was due to an increase in transcript stability and not increased promoter activity. Further, in another study it has been demonstrated that accumulation of several cold-inducible transcripts occurred due to post-transcriptional events [49].

In summary, measurement of relative rate of gene transcription with this protocol revealed the important matrix of promoter activity and transcript stability of TDC gene in response to external cue. This information would be valuable in investigating the transcriptional and post transcriptional response of other TIA pathway genes in *C roseus*. The results presented in this work propose an efficient method for the isolation of intact *C*. *roseus* nuclei which could be used across different plant tissues and plant growth conditions. The measurement of relative rate of gene transcription with this protocol revealed the important matrix of promoter activity and gene expression or regulation. Isolated nuclei may also provide a resource that could be used for performing the chip assay as well as serve as the source of nuclear proteins for *in-vitro* EMSA studies. Moreover, nascent nuclear run-on transcript could be further subjected to RNA-Seq for global nuclear run-on assay (GNRO-Seq) for genome wide *in-situ* measurement of transcription rate of plant genes.

## Supporting Information

S1 Table List of Primers used in this study. (DOC)
