# Supplementary material for: Isolation of Catharanthus roseus (L.) G. Don Nuclei and Measurement of Rate of Tryptophan decarboxylase Gene Transcription Using Nuclear Run-On Transcription Assay
Source: PLoS One. 2015 May 29;10(5):e0127892. doi: 10.1371/journal.pone.0127892 (PMC4449189; doi:10.1371/journal.pone.0127892)
Supplement: S1 Table — (DOC) [file pone.0127892.s001.doc]

**S1 Table. List of Primers used in this study**

| **S.N.** | **Name** | **Gene ID** | **Primer Sequences for blotting** | **Primer Sequences for qRT-PCR** | **Reference** |
| --- | --- | --- | --- | --- | --- |
| 1 | SAND | Caros010066.1 | Fw- TCTCCCCAATTTGCCTTCCC  Rv- TTGAGTGACCCAGCAAAGCA | Fw- GCTGTGGAGGAGGAAGAAG  Rv- ACTGGCGGAACTACTACTACC | Pollier et al. 2014 |
| 2 | N227 | Caros011588.1 | Fw- CTCGAAGTGGCCCTCGAAAT  Rv- GCAACTGCAACGAAGGATCC | Fw- CCTTACGCCGCATTATCAG  Rv- AGATGAGACAGTAACGCCTTG | Pollier et al. 2014 |
| 3 | EXP | Caros010480.1 | Fw- CAATCCACCGACTCTGCCAT  Rv- GCCTCCCTCCCAGGAATTTC | Fw-ACAATACCATCGCCATCAC  Rv- AAGAGGACTGCTGGAAGG | Pollier et al. 2014 |
| 4 | RPS9 | Caros004092.1  /AJ749993 | Fw- GATCGCCGAGCTGAAGATCA  Rv- CCATCACCACCAGATGCCTT | Fw-GTTGTCAATGTTCCTTCCTTC  Rv- TCTTCATCCTCTTCATCTCCATC | Pollier et al. 2014 |
| 5 | TDC | M25151.1 | Fw- GAAACCGCTCCTTACCTCCC  Rv- GCATAAGCAGCATCCACGTG | Fw- AGCGAAGTCGAACCTGGATATC  Rv- GGGAGGTAAGGAGCGGTTTC |  |
| 6 |  | AF253415.1 | Fw- TCCATTTGCTGGAAAAGTTG  Rv-CGAACATCTACAAATTCAATT |  |  |
